# Commentary: Burst Firing in a Motion-Sensitive Neural Pathway Correlates with Expansion Properties of Looming Objects That Evoke Avoidance Behaviors

**DOI:** 10.3389/fnint.2015.00068

**Published:** 2016-01-11

**Authors:** Jessica L. Fox

**Affiliations:** Case Western Reserve UniversityCleveland, OH, USA

**Keywords:** sensory coding, bursting, vision, locust, Neuron, DCMD

What is the neural code? This essential question has been the driving force behind much research in sensory and motor neuroscience, spurring investigations of diverse animals, brain areas, and behaviors. Given that neurons generally transmit information with trains of voltage spikes, what information are these spikes representing, and how can they be interpreted? Do downstream neurons respond to spike rates, counts, times, or some combination of these parameters? How does changing the pattern of spikes change behavior? These are fundamental questions in computational and behavioral neuroscience, and the answers have been as diverse as the neurons themselves.

The reflexive response of locusts to looming stimuli is a classic model in neuroethology, with a small number of neurons encoding the expanding stimulus and with spike trains resulting in a robust jump response or wing steering maneuvers. Because the neural circuit is relatively direct, from retina to muscle in only a handful of synapses, it is an excellent candidate for the study of neural coding. In this pathway, the visual expansion of a dark circle on the locust's retina, representing a looming threat, results in a train of spikes in the lobula giant motion detector (LGMD) that signal to a descending interneuron, the descending contralateral motion detector (DCMD) (Gabbiani et al., 1999). The DCMD then stimulates thoracic interneurons and motoneurons to initiate jump or wing-steering responses (Burrows and Fraser Rowell, 1973). In previous work, various parameters of the DCMD spike train, including firing rate, time of peak firing rate, and total spike count, were found to control different aspects of the jump response (Fotowat et al., 2011), indicating that a train of spikes from a single neuron can modulate responses in multiple ways. This multiplexing suggests that the DCMD spike train is a powerful means of motor control.

Previous analysis of the DCMD response to a looming stimulus found that spike rate increases as the stimulus expands, peaking within 200 ms of the predicted collision and peaking sooner when the stimulus is moving faster. In a new study, McMillan and Gray analyzed DCMD spike trains looking for evidence of neural bursting, which they defined using specific statistics, most importantly an inter-spike interval of <8 ms. They found that the DCMD neuron does indeed burst, and most of these bursts occur when the looming stimulus becomes imminent (>200 ms before the predicted collision time). By separating the spikes in bursts from isolated spikes, they found that the increase in spike rate that occurs in the 200 ms before collision is due almost entirely to an increase in bursting, not an increase in the rate of single spikes.

While the increase in bursting is intriguing, the burst activity is only of importance if it encodes some information about the stimulus. In a related insect, the cricket, burst responses of auditory neurons encode high-frequency bat calls and evoke evasive responses, while isolated spikes encode communication signals from conspecifics (Marsat and Pollack, 2006). The bursts in this neuron are powerful signifiers that result in specific and stereotyped behaviors, whereas the isolated spikes play a different role. Furthermore, two spikes in a burst carry significantly more information than two isolated spikes (Brenner et al., 2000), and thus the bursts may be sending a very specific and important signal to downstream neurons and muscles. What does bursting in the DCMD signify? To gain some clues, McMillan and Gray re-analyzed a previous data set, in which they varied the speed of the looming stimulus, and applied their burst-detection algorithm. They found that the peak firing rate of isolated spikes does not change with the stimulus velocity, but the peak firing rate of the spikes in bursts does. Similarly, firing rate of the bursts and of the spikes within the bursts peaked later when the stimulus moved faster, while the time of peak firing rate of isolated spikes did not change when stimulus velocity was varied. Both phenomena could be a result of isolated spikes clustering themselves into bursts as the stimulus looms closer, but McMillan and Gray's analysis shows that encoding of the stimulus is not just a matter of an increase in the rate of single spikes, but rather of distinct bursts appearing in the neural code at key times in a behaviorally-relevant stimulus paradigm. The bursts appear around 200 ms before collision, and do not increase their rate when the stimulus moves faster. The spikes within the bursts, however, arrive at an increased rate when stimulus velocity is increased. As this is occurring, firing rate of the isolated spikes decreases, and the amount of this decrease is negatively correlated with the stimulus velocity. Thus, the presence of bursts, which arrive at a rate similar to the locust's wingbeat (~30 Hz) and do not modulate their rate with speed, indicates that the looming stimulus is present. The speed of the looming stimulus is encoded secondarily, with spikes in the bursts arriving faster when the stimulus is moving faster. By examining spike trains in greater detail, McMillan and Gray discovered that the DCMD is using *both* a timing code and a rate code to indicate the presence and speed of a threatening stimulus.

The new-found complexity of the DCMD neural code is intriguing, but is bursting activity linked to evasive behavior? Future experiments will be needed to determine if the bursting activity in DCMD can modulate escape jumps or wing steering. Given that adding spikes to the DCMD train results in wing-steering asymmetry (McMillan et al., 2013), the current study suggests that bursting activity might result in modulations of aerial turns. By encoding multiple stimulus parameters with different parameters of the spike response, the DCMD would be able to stimulate complex patterns of behavior without requiring the downstream motor neurons to make substantial neural computations of their own. Using bursts and single spikes independently, stimulus information is parsed into two channels that can impact the motor system differently in the face of an oncoming threat, potentially endowing the escape circuit with both speed and flexibility.

## Conflict of interest statement

The author declares that the research was conducted in the absence of any commercial or financial relationships that could be construed as a potential conflict of interest.
